# A narrative review of adaptive Phase I/II clinical trial designs for drug combinations in oncology

**DOI:** 10.3389/fonc.2026.1916456

**Published:** 2026-09-21

**Authors:** Junying Wang, Song Wu, Jie Yang

**Affiliations:** 1 Department of Anesthesiology, Critical Care and Pain Management, Hospital for Special Surgery, New York, NY, United States; 2 Department of Applied Mathematics and Statistics, Stony Brook University, Stony Brook, NY, United States; 3 Department of Family, Population and Preventive Medicine, School of Medicine, Stony Brook University, Stony Brook, NY, United States

**Keywords:** adaptive dose-finding design, adaptive randomization, Bayesian adaptive design, narrative review, oncology trials, Phase I/II adaptive design for drug combinations, seamless design

## Abstract

**Background:**

Adaptive Phase I/II designs can jointly use toxicity and efficacy information to guide dose allocation and regimen selection. These designs are particularly relevant for oncology drug-combination trials, where the optimal regimen may not coincide with the maximum tolerated dose.

**Methods:**

We conducted a structured narrative review of adaptive Phase I/II designs for oncology drug combinations that use both toxicity and efficacy information for adaptive allocation or final regimen selection. PubMed was searched through April 16, 2026 using terms related to Phase I/II, seamless design, drug combinations, dose-finding and efficacy, optimal biological dose, dual-agent, efficacy-toxicity trade-off, utility-based dose-finding, and adaptive randomization. Google Scholar and reference lists were used for supplementary citation chasing, and ClinicalTrials.gov was searched for illustrative real-trial applications.

**Results:**

Adaptive Phase I/II designs for drug combinations were identified across model-based, model-assisted, utility-based, and response-adaptive randomization frameworks for both discrete and continuous dose settings. Across designs, the main trade-offs involved assumptions on monotonicity and ordering, the ability to handle delayed outcomes, transparency of decision rules, computational burden, and software availability. Our targeted search identified few published real-world implementations, and software directly tailored to seamless Phase I/II combination trials remained limited.

**Conclusions:**

Adaptive Phase I/II designs for drug combinations are methodologically well developed but remain difficult to implement routinely. Broader use will require clearer design-selection guidance, accessible software, robust delayed-outcome extensions, and protocol templates that communicate operating characteristics to clinical and regulatory stakeholders.

## 1 Background

Adaptive clinical trial designs permit prospectively planned modifications based on accumulating trial data and have become increasingly important in oncology, where rapid learning about benefit-risk trade-offs is often essential. In early-phase oncology trials, these designs can improve efficiency, reduce exposure to ineffective regimens, and allow earlier stopping for safety, futility, or strong efficacy signals (1–4).

Seamless Phase I/II designs are especially attractive when the objective extends beyond identifying the maximum tolerated dose and requires joint consideration of toxicity and efficacy. This is particularly relevant for contemporary oncology development, where the most clinically useful dose may be an optimal biological dose rather than the maximum tolerated dose, and where treatment effects may emerge before conventional phase transitions would normally occur (4–6).

Drug-combination trials add another layer of difficulty. Compared with single-agent dose-finding, combination studies must contend with partial ordering of dose pairs, potential drug-drug interactions, a larger design space, and the practical challenge of balancing two outcomes while treating only a limited number of patients. These issues are amplified in oncology, where combinations are often evaluated to improve efficacy, delay resistance, or exploit complementary mechanisms of action (7–9).

A number of seamless Phase I/II methods have been proposed for oncology drug combinations, but they differ substantially in dose scale (discrete versus continuous), endpoint structure (binary, ordinal, or continuous efficacy), modeling assumptions, adaptive allocation strategy, handling of delayed outcomes, and software availability. As a result, trialists may recognize the names of existing designs without having a clear sense of when each design is most appropriate or what practical trade-offs each requires.

The purpose of this article is to provide a structured narrative overview of in-scope methods for oncology drug combinations that simultaneously evaluate toxicity and efficacy. Rather than introducing a new methodology, we provide a comprehensive review of existing designs, identifying their assumptions and operational features, highlighting practical strengths and limitations, reviewing published real-trial applications, and describing the current software landscape. We focus specifically on the simultaneous Phase I/II approach and do not consider sequential phase-I-then-Phase II programs, except where needed for context.

## 2 Methods

This article is a structured narrative methodological review rather than a formal systematic review. The aim is to systematically map and compare available adaptive Phase I/II designs for oncology drug combinations, rather than to estimate pooled treatment effects or perform a quantitative synthesis of evidence.

PubMed was searched from database inception through April 16, 2026 using combinations of the following terms: “Phase I/II”, “seamless”, “drug combination”, “dual-agent”, “dose-finding and efficacy”, “optimal biological dose”, “efficacy-toxicity trade-off”, “utility-based dose finding”, and “adaptive randomization”. Google Scholar was used only as a supplementary source for forward citation chasing and for identifying methodological papers, software pages, and author-supplied code not indexed in PubMed. ClinicalTrials.gov was searched to identify published oncology trials that appeared to have implemented in-scope seamless Phase I/II combination designs.

The literature search identified 195 records. After title and abstract screening, 19 articles were assessed for full-text eligibility review, of which 11 methodological articles met the predefined eligibility criteria and were included in this review. We included articles that proposed methodologies for adaptive Phase I/II designs for drug combinations with simultaneous consideration of toxicity and efficacy. We excluded articles that focused exclusively on single-agent development, Phase I combination designs evaluating toxicity alone, and two-stage programs that assessed toxicity in Phase I and efficacy in a separate Phase II stage without joint estimation. For clarity, we considered a design to be seamless or involving joint estimation if a single patient population spanned both stages and efficacy information informed, or was integrated into, toxicity-based allocation or regimen selection (e.g., information borrowing across stages). Two-stage continuous-dose designs that allocate for efficacy along a stage-1 maximum-tolerated-dose curve within one patient population meet this criterion, whereas purely sequential programs that finalize toxicity in a separate Phase I cohort before an independent Phase II were not considered seamless designs.

For each included design, we extracted the dose scale (discrete or continuous), endpoint type, key modeling assumptions, adaptive allocation strategy, approach to delayed outcomes, software availability, and evidence of clinical implementation. Because this was a narrative methodological review rather than a systematic review of empirical outcomes, formal risk-of-bias assessment, PRISMA flow reporting, and meta-analysis were not undertaken. To enhance transparency, however, we report the search sources and date, eligibility criteria, and data abstraction domains.

## 3 Phase I/II adaptive designs for drug combinations

We grouped the identified methods according to dose-scale type (discrete versus continuous) and by endpoint structure, as these features largely determine how toxicity-efficacy trade-offs are modeled and how patients are adaptively allocated. In addition to describing the core statistical framework of each design, we highlight the practical settings in which each approach is most applicable. The identified designs are summarized in **Table 1**.

**Table 1:** Comparative summary of Phase I/II adaptive designs for drug combinations.

Paper (Year)	Dose type	Toxicity and efficacy endpoint types	Key assumptions	Recommended use	Adaptive method	Delayed outcome handling?	Software
Yin, Li, and Ji (10) (2006)	Discrete	Bivariate binary outcome	Monotonic toxicity; No constraints for dose-response curve	Joint modeling of correlated toxicity and efficacy outcome	Bayesian adaptive method	No delayed-outcome handling	Not reported
Huang et al. (11) (2007)	Discrete	Both binary outcomes	No specified assumptions	Simple discrete combination trials with adaptive randomization after initial safety assessment	Modified 3 + 3 for dose combinations; Response-adaptive randomization from Bayesian posterior efficacy probabilities	No delayed-outcome handling	Not reported
Yuan and Yin (12) (2011)	Discrete	Both binary outcomes	Copula-type toxicity model; monotonic toxicity; efficacy modeled adaptively among admissible doses	Discrete combination trials with adaptive randomization among safety-admissible dose combinations	Bayesian copula-type model for Phase I dose-finding design; Response-adaptive randomization	No delayed-outcome handling	Code is available upon request.
Wages and Conaway (13) (2014)	Discrete	Both binary outcomes	Monotonically increasing dose-toxicity and dose-efficacy curves with partial orderings by each drug	Combination trials where partial ordering among dose combinations can be specified	CRM Phase I dose-finding design; Response-adaptive randomization	Delayed efficacy accommodated (no time-to-event updating)	Code is available upon request.
Thall et al. (14) (2017)	Discrete	Both four-level ordinal outcomes	Generalized continuation ratio model for each marginal distribution; Copula model for bivariate outcomes with dimensions (4,4)	Trials requiring explicit benefit–risk optimization using ordinal toxicity and efficacy outcomes	Bayesian adaptive design; Response-adaptive randomization	No delayed-outcome handling	A computer program named ‘U2OET’ can be downloaded from: https://biostatistics.mdanderson.org/SoftwareDownload
Mozgunov and Jaki (15) (2019)	Discrete	Both binary outcomes	No parametric or monotonicity assumptions	Efficacy may be non-monotonic and benefit–risk trade-offs are prioritized	Novel efficacy-toxicity trade-off function; Response-adaptive randomization	Delayed efficacy accommodated (no time-to-event updating)	No public software identified.
Mozgunov and Jaki (16) (2019)	Discrete	Binary toxicity and continuous efficacy outcomes	No parametric or monotonicity assumptions; Logistic transformation to map continuous efficacy from (−∞, +∞) to (0, 1)	Trials with continuous efficacy endpoints where efficacy may not increase monotonically with dose	Novel efficacy-toxicity trade-off function; Response-adaptive randomization	Delayed efficacy accommodated (no time-to-event updating)	https://github.com/dose-finding/cont-phase-I-II
Tighiouart (17) (2019)	Continuous	Both binary outcomes	Monotonic toxicity for each drug; Efficacy optimized along the estimated MTD curve	Early-phase continuous-dose combination trials that first estimate an MTD curve before optimizing efficacy along that curve	EWOC dose-finding design; Bayesian adaptive design; Response-adaptive randomization	Related time-to-event extension available	No public software identified
Jiménez and Tighiouart (18) (2022)	Continuous	Both binary outcomes	Optimal dose combination located along the MTD curve; Monotonic toxicity and efficacy	Continuous combination trials requiring an adaptive Phase I/II design with adaptive randomization along the estimated MTD curve.	EWOC dose-finding design; Response-adaptive randomization	Related time-to-event extension available	https://github.com/jjimenezm1989/Phase-I-II-design-combining-two-cytotoxics
Jiménez and Zheng (19) (2023)	Continuous	Both binary outcomes	Monotonic toxicity and efficacy	Continuous combination trials where borrowing efficacy information across Phase I and Phase II is desirable to improve estimation efficiency.	EWOC dose-finding design; Bayesian adaptive method	Related time-to-event extension available	No public software identified
Wang et al. (20) (2023)	Discrete	Both binary outcomes	Monotonic toxicity; multiple candidate dose-efficacy models for either monotonic or non-monotonic efficacy	Trials where the dose–efficacy relationship is uncertain and adaptive Bayesian model selection is desired	Bayesian adaptive model selection	Delayed efficacy accommodated (no time-to-event updating)	No public software identified

*Supplementary Table 1 provides more detailed technical comparisons of the specific adaptive methods used.

### 3.1 Discrete dose levels for drug combinations

#### 3.1.1 Binary endpoints for toxicity and efficacy

Huang et al. (11) proposed a parallel Phase I/II clinical trial to simultaneously evaluate efficacy and toxicity and select the optimal combination dose of two drugs. In Phase I, they implement the modified “3 + 3” design (21), and in Phase II, they employ adaptive randomization guided by Bayesian posterior probabilities of response rate to allocate more patients to dose levels with better efficacy. The design incorporates early stopping rules based on predefined criteria for toxicity, efficacy, and futility. Dose combinations falling below the acceptable efficacy threshold or exhibiting intolerable toxicity are removed from further evaluation. The reported simulations suggest that the design may reduce the required sample size while improving the probability of identifying the most efficacious treatment arms and allocating a greater proportion of patients to the most effective dose combinations.

Yuan and Yin (12) proposed an adaptive Phase I/II design for combination trials aimed at identifying the most efficacious dose combination among those that satisfy prespecified safety requirements. In Phase I of this method, a Bayesian copula-type dose-finding model is employed to identify a set of admissible doses that meet the safety criteria. The copula-type model estimates the toxicity probability *π_ij_*, for the combination of two drugs, A and B, as:


$$\pi_{ij}=1-{\left[{\left(1-a_{i}^{\alpha }\right)}^{-\gamma }+{\left(1-b_{j}^{\beta }\right)}^{-\gamma }-1\right]}^{-1/\gamma },$$

where *α*, *β*, *γ* > 0 are unknown model parameters with gamma prior distributions, determined through the posterior density within a Bayesian framework. The parameters *a_i_*, *i* = 1,…, *I* and *b_j_*, *j* = 1,…, *J* are prespecified toxicity probabilities for two drugs, respectively. The resulting set of admissible dose combinations is then carried forward into Phase II, where an innovative response-adaptive randomization strategy allocates more patients to the most efficacious dose combinations. The randomization schema uses a moving reference to compare the efficacy of the admissible doses found in Phase I. In the simulations reported by the authors, this method effectively distinguished among treatment arms under the evaluated scenarios.

Wages and Conaway (13) developed a method that assumes a partial ordering of toxicity probabilities across drug combinations, and uses CRM to guide patient allocation (22). This approach jointly evaluates toxicity and immunologic response, with the latter serving as the measure of efficacy. For each order *m* among a total of *M* orderings, the true probability of DLT response at dose combination *d_i_* among *I* dose combinations is modeled as a power function, $\pi_{T}\left(d_{i}\right)=P\left[X=1|d_{i}\right]\approx F_{m}\left(d_{i},\;\beta_{m}\right)=p_{m_{i}}^{\text{exp}\left(\beta_{m}\right)}$, where $\beta_{m}\in \mathbb{R}$ is the parameter estimated from the data and $p_{m_{i}}$ are prespecified toxicity probabilities between 0 and 1, often referred to as the “skeleton”, which are chosen either from clinical experience or using the algorithm proposed by Lee and Cheung (23). For each *m*, the maximum likelihood estimate ${\hat{\beta }}_{m}$ of the parameter *β_m_* can be obtained by maximizing its likelihood function, $ℒ\left({\hat{\beta }}_{m}\right)$. To guide decision-making, one method is to assign a weight *ω*(*m*) to each ordering *m* by


$$\omega \left(m\right)=\frac{ℒ\left({\hat{\beta }}_{m}\right)\xi \left(m\right)}{\sum_{k=1}^{M}ℒ\left({\hat{\beta }}_{k}\right)\xi \left(k\right)},$$

where *ξ*(*m*) satisfies ∑ξ(m)=1 and the weights are used as the ordering probabilities for the next enrolled patient. If no prior information for the ordering is available, a discrete uniform distribution with $\xi \left(m\right)=\frac{1}{M}$ can be specified. When enrolling a new patient, a single model m^*^ is chosen, and the DLT probabilities ${\hat{\pi }}_{T}\left(d_{i}\right)$ are estimated at each regimen by ${\hat{\pi }}_{T}\left(d_{i}\right)=F_{m^{*}}\left(d_{i},\;{\hat{\beta }}_{m^{*}}\right)$, where $m^{*}=\arg\max_{m}\omega \left(m\right)$. The acceptable set A is defined as the dose combinations with toxicity probability smaller than a prespecified threshold. Similarly, a dose-efficacy probability $\pi_{E}\left(d_{i}\right)=q_{k_{i}}^{\text{exp}\left(\theta_{k}\right)}$ is defined using a power function for each efficacy order *k* and parameter *θ_k_* with prespecified efficacy probabilities (skeleton) $q_{k_{i}}$. The recommended regimen for the next patient, *d_i_*, is selected from the acceptable set A with the highest immune response rate $d_{i}=\arg\max_{d_{i}\in A}{\hat{\pi }}_{E}\left(d_{i}\right)$. In practice, the design averages over plausible toxicity orderings, restricts allocation to combinations with acceptable estimated toxicity, and then selects among acceptable combinations using estimated immune-response probability.

To avoid parametric or monotonicity assumptions, Mozgunov and Jaki (15) proposed a Phase I/II design that identifies the optimal dose using a derived efficacy-toxicity trade-off function. The authors developed dose-finding algorithms for both monotonic and non-monotonic relationships between toxicity and efficacy, and extended the approach to accommodate delayed efficacy responses when both toxicity and efficacy are treated as binary endpoints.

More recently, Wang et al. (20) proposed a Phase I/II dose-finding design to identify the optimal dose combination by maximizing the posterior mean of a utility function. This utility function assigns pre-defined scores to each possible combination from binary outcomes of toxicity and efficacy. Let $x_{a,j},\;j=1,\ldots ,J$ denote the J dose levels of drug A, and $x_{b,k},\;k=1,\ldots ,K$ for the *K* dose levels of drug B, where *J* ≥ *K*. The doses are standardized with a mean of 0 and a standard deviation of 0.5. Then, let $p(x_{jk})$ and $q(x_{jk})$ be the toxicity and efficacy probabilities of the combinations of drugs $x_{jk}=\left(x_{a,j},x_{b,k}\right)$. The dose-toxicity curve is modeled as a logistic function with three parameters: $\text{logit }(p(x_{jk}))\;=\;\alpha_{0}+\;\alpha_{1}x_{a,j}+\;\alpha_{2}x_{b,k}$, where $\alpha ={\left(\alpha_{0},\;\alpha_{1},\;\alpha_{2}\right)}^{\prime }$ is the parameter vector representing the intercept and slopes for two drugs. For dose-efficacy relationships, three distinct logistic models are considered: the first one focuses solely on the main effects of the two drugs, similar to the dose-toxicity curve; the second one adds an interaction term between two drug doses; and the third one further adds quadratic terms for main effects for each drug dose. Then an adaptive Bayesian model selection is applied to choose the efficacy model closest to the dose-response surface. The main advantage of this design lies in its flexibility regarding the dose-efficacy relationship. Unlike approaches based on a single prespecified efficacy model, it allows the estimated dose–response relationship to adapt to either monotonic or non-monotonic shapes in response to newly accumulating data, thereby accommodating a broader range of dose-efficacy scenarios. By moving away from a single-model constraint, this approach may reduce the effect of model misspecification on the trial’s operational characteristics, allowing for a more nuanced and responsive evaluation of treatment effects.

#### 3.1.2 Other types of endpoints for toxicity and efficacy

Yin, Li, and Ji (10) developed a Phase I/II design that jointly assesses toxicity and efficacy for a new treatment. The authors treat toxicity and efficacy as correlated bivariate binary outcomes, using the odds ratio - calculated from the marginal probabilities of toxicity and efficacy - as a measure with a small value indicating higher efficacy with lower toxicity. In addition, they introduce an alternative three-dimensional volume ratio to evaluate the correlation, and the three dimensions include toxicity, efficacy, and efficacy given no toxicity. This nonparametric design is not influenced by how the tested dose combinations would be selected. However, it needs a comprehensive evaluation of both toxicity and efficacy for each patient cohort before determining the subsequent cohort’s allocation, which could prolong the duration needed to collect and analyze all response data.

Thall et al. (14) presented a Phase I/II design for a two-drug combination that employs four-level ordinal outcomes to assess both efficacy and toxicity. The optimal combination of the two agents is determined by the largest posterior mean utility, derived from a Bayesian model that assigns prespecified numerical utilities to each of the 16 efficacy-toxicity outcome pairs. Response-adaptive randomization, which selects dose pairs based on the calculated posterior mean utilities, is used to reduce the risk of becoming concentrated at a suboptimal dose pair.

Mozgunov and Jaki (16) extended their method from binary toxicity and efficacy endpoints (15) to binary toxicity and continuous efficacy endpoints. The continuous efficacy outcome is incorporated into the escalation step by transforming the efficacy endpoint to the unit interval with an inverse logistic function.

### 3.2 Continuous dose levels for drug combinations

#### 3.2.1 Binary endpoints for toxicity and efficacy

Tighiouart (17) presented a two-stage Phase I/II design that deals with continuous dose levels and evaluates treatment efficacy for three or more cycles of therapy to select a dose combination with the maximum probability of response. In Phase I, the method of Tighiouart et al. (24) is used to estimate the MTD curve that lies within a Cartesian plane defined by the ranges of the two continuous doses, with cubic splines modeling the dose-response relationship for various functional forms of the efficacy curve. In Phase II, a Bayesian adaptive design is used to allocate patients along the MTD curve obtained in Phase I to determine a drug combination with the maximum probability of treatment efficacy.

Jiménez and Tighiouart (18) refined the two-stage Phase I/II design for combinations of two cytotoxic agents with continuous dose levels. They incorporated the assumption that a single patient population could span both phases. In Phase I, they implement the EWOC method to obtain the estimated MTD curve and use response-adaptive randomization to assign patients according to the MTD curve. Prespecified toxicity and efficacy targets, together with Bayesian posterior updating, guide MTD estimation, Phase II efficacy evaluation, optimal-dose selection, and early stopping for futility or safety. Similar to the settings used in Tighiouart (17), the target probability of DLT, *θ_Z_*, and efficacy of the standard treatment, *θ_E_*, must be prespecified [e.g. $\theta_{Z}=0.33$ and $\;\theta_{E}=0.15$ used in their simulation studies (18)]. The goal is to identify, along the estimated MTD curve, the drug combination (*x*, *y*) that satisfies the target DLT constraint, $\pi_{Z}\left(x,y\right)\leq \theta_{Z}$ and maximizes the probability of treatment response, $\pi_{E}\left(x,y\right)\geq \theta_{E}$. A non-informative Jeffreys prior beta(0.5,0.5) can be applied to the DLT rate Θ in both phases regardless of dose combinations.

Jiménez and Zheng (19) introduced a phase-specific efficacy model that separates the contributions of the individual drugs and their interaction. A robust Bayesian hierarchical model is used to combine efficacy data across two phases, which improves the precision of parameter estimation and reduces the number of patients treated at subtherapeutic dose combinations. Additional technical details about these designs are provided in **Supplementary File 2**.

### 3.3 Practical guidance for design selection

The reviewed designs have the common objective of simultaneously evaluating toxicity and efficacy; however, the operating characteristics reported in the original publications were obtained under different dose grids, sample sizes, toxicity and efficacy scenarios, endpoint definitions, and performance criteria. Consequently, measures such as correct-selection probability, overdose probability, expected utility, and allocation to the optimal regimen are not directly comparable across studies. Accordingly, the comparisons presented in this review are qualitative and based on the underlying assumptions, methodological features, and intended application of each design, rather than on head-to-head performance rankings. No single design is universally optimal for all oncology drug-combination trials. Instead, the choice of design should be guided by the trial objectives, endpoint characteristics, assumptions regarding dose-response relationships, and practical considerations such as delayed outcomes and software availability. To facilitate design selection, **Figure 1** provides a simplified selection framework for the major design families, and **Table 1** summarizes the key characteristics and recommended use of each design. The following section synthesizes the principal factors investigators should consider when choosing an adaptive Phase I/II design.

**Figure 1:**
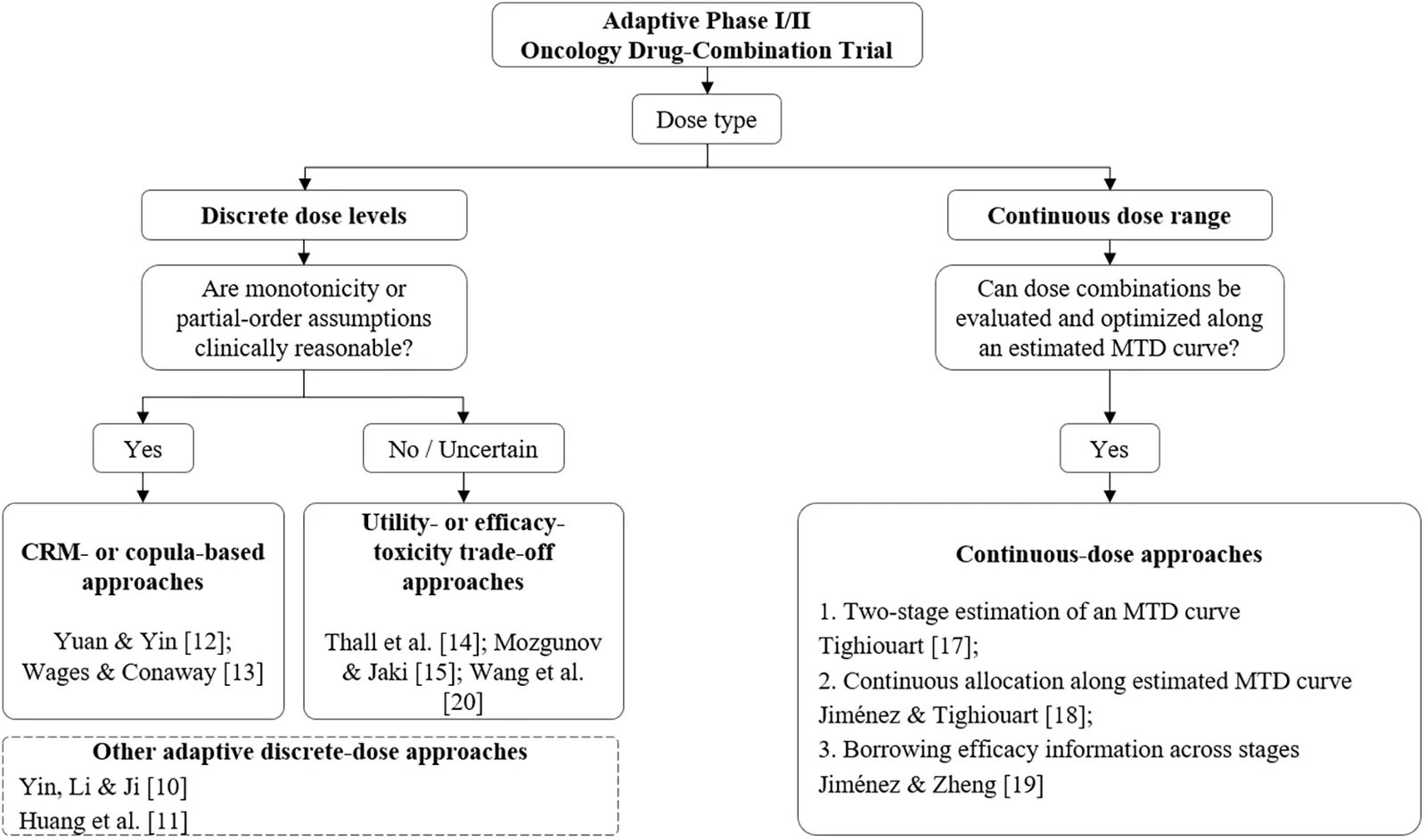
Practical framework for selecting adaptive Phase I/II drug combination designs based on dose scale and structural dose-response assumptions.

Across these design families, several practical patterns emerge that may guide investigators in selecting an appropriate adaptive Phase I/II design (**Figure 1**). Design choice should be driven by the clinical setting, dose structure, and trial objectives rather than by the assumption that one methodology is generally superior. Parallel Phase I/II designs and simpler response-adaptive randomization rules are relatively transparent, although they may use accumulating information less efficiently. CRM- or copula-based combination designs are attractive when investigators can reasonably justify monotonicity or partial-order assumptions, whereas utility- or trade-off-based designs are more appealing when the optimal biological dose is not expected to coincide with the maximum tolerated dose. Continuous-dose methods can be useful when dose pairs can be tuned along an estimated MTD curve, but they generally require stronger modeling assumptions and greater implementation effort. Investigators should also consider practical implementation factors, such as software availability, computational and operational complexity, and the potential for delayed toxicity or efficacy outcomes, particularly because relatively few adaptive Phase I/II combination designs currently accommodate true time-to-event updating. Because operating characteristics reported for individual designs are based on different simulation scenarios, they are not directly comparable across methods, and relative performance should not be inferred from them.

Another important consideration is the quantity of prior knowledge available for each investigational agent. When one agent has been well characterized in previous single-agent studies and the other is novel, historical information on the established agent, such as toxicity profile, effective dose range, and prior dose-response relationship, may be used to inform prior distributions, CRM skeleton calibration, admissible dose combinations, or utility specification, thereby potentially improving trial efficiency. Because combination therapy may introduce synergistic or antagonistic effects on both toxicity and efficacy, single-agent operating characteristics should not be assumed to translate directly to the combination setting. When both agents are novel and limited prior information is available, more conservative adaptive approaches that rely less heavily on informative priors and emphasize prospective learning of the joint toxicity-efficacy profile may be preferable. Regardless of the development scenario, historical information is best used to guide prior specification and simulation during trial planning, whereas dose-allocation decisions should remain primarily driven by accumulating combination-specific data.

A further limitation across the reviewed literature is the relatively limited accommodation of delayed efficacy and toxicity outcomes in combination trials. It is also important to note that these continuous-dose designs locate the optimal combination on the estimated MTD curve under an assumption of a monotonic dose–efficacy relationship. This suits cytotoxic combinations but is structurally mismatched to targeted agents or immunotherapies, where efficacy may plateau or be non-monotone and the optimal biological dose can lie below the MTD surface. In such settings, a design that searches the full dose grid is preferable.

## 4 Real trial examples

ClinicalTrials.gov was searched on April 16, 2026 using terms including “Phase I/II,” “adaptive,” “dose-finding,” “combination,” and “oncology”. A real-trial example was considered in scope if the published protocol or methods paper explicitly described adaptive dose allocation or final regimen selection using both toxicity and efficacy information in a drug-combination Phase I/II framework. Our targeted search identified two published oncology trials that clearly met the inclusion criteria. This limited uptake is itself informative and likely reflects practical barriers rather than a lack of methodological developments. Such barriers include the need for bespoke programming, limited availability of practical software tools, the difficulty of communicating complex adaptive rules to multidisciplinary clinical teams, and operational or regulatory caution when both toxicity and efficacy inform dose allocation decisions. Together, these challenges increase the statistical and operational burden of implementing seamless adaptive combination designs, thereby limiting their broader adoption in routine clinical practice. We therefore present the available real-trial examples not only as illustrations of design use, but also as evidence of the translational gap between methodological development and practical implementation.

One notable example is the multicenter clinical trial by Wages et al. (25), titled “Phase I/II Trial of a Long Peptide Vaccine (LPV7) Plus TLR Agonists” (MEL60, ClinicalTrials.gov Identifier: NCT02126579), designed at the University of Virginia Cancer Center. This trial evaluated a long-peptide vaccine (LPV7) plus TLR agonists for patients with resected stage IIB–IV melanoma. The design was based on CRM with simultaneous consideration of binary toxicity and immune-response efficacy. In Stage 1, patients were accrued until a DLT event occurred to stabilize toxicity estimation. In Stage 2, each new patient was allocated to the acceptable regimen with the highest estimated response probability. This example shows that joint toxicity-efficacy learning is feasible in practice, but it also highlights the level of disease-specific tailoring required for implementation.

Wages et al. (26) applied a closely related methodology in another melanoma trial titled “Safety Study of a Helper Peptide Vaccine Plus Adjuvant Combinations for the Treatment of Melanoma” (Mel63; CHAMP, ClinicalTrials.gov Identifier: NCT02425306). The modified allocation rule combined weighted randomization early in the trial with deterministic assignment to the currently best acceptable regimen later in the trial. Together, these two trials suggest that in-scope adaptive Phase I/II combination designs can be implemented clinically. However, because both examples arose from the same methodological lineage, they also underscore the limited clinical uptake of alternative seamless Phase I/II combination designs.

## 5 Software tools

Software that directly implements the seamless Phase I/II combination designs reviewed in Section 3 remains limited. **Table 2** therefore distinguishes between software that directly implements, or closely approximates, in-scope Phase I/II designs and adjacent early-phase packages that are useful for Phase I combination trials or single-agent Phase I/II settings but do not directly solve the seamless combination problem.

**Table 2:** Selected software for early-phase clinical trials, including adjacent tools that contextualize the software gap for seamless Phase I/II combination designs.

Package	Version	Last updated	Phase	Drug type	Toxicity/Efficacy	Directly supports Phase I/II drug combinations?
CRM (27)	1.2.4	2018-09-26	Phase I	Single Drug	Toxicity	No
dfcrm (28)	0.2-2.1	2019-01-26	Phase I	Single Drug	Toxicity	No
crmPack (29)	2.1.1	2026-07-27	Phase I/II	Single Drug, Drug combinations	Toxicity/Efficacy	Partial; does not simultaneously estimate toxicity and efficacy
bcrm (30)	0.5.4	2019-08-23	Phase I	Single Drug	Toxicity	No
trialr (31)	0.1.6	2023-03-12	Phase I	Single Drug	Toxicity/Efficacy	No
ewoc (32)	0.3.0	2020-06-07	Phase I	Single Drug	Toxicity	No
BOIN (33)	2.7.2	2021-01-19	Phase I	Drug combination	Toxicity	No
pocrm (34)	0.13	2021-09-08	Phase I	Drug combination	Toxicity	No
MinEDfind (35)	0.1.3	2020-05-05	Phase II	Single Drug	Efficacy	No
clinfun (36)	1.1.5	2023-10-19	Phase I/II	Single Drug	Toxicity/Efficacy	No
crm12Comb (37)	0.1.12	2026-02-03	Phase I/II	Drug combination	Toxicity/Efficacy	Yes
dfcomb (38)	3.1-5	2026-03-02	Phase I/II	Drug combination	Toxicity/Efficacy	Yes

(1) Some packages do not directly implement Phase I/II drug-combination designs but are included because they support related early-phase designs. All listed R packages use discrete dose levels unless otherwise noted.

(2) Binary endpoints were considered for toxicity and efficacy for all R packages except that crmPack allows continuous efficacy endpoints, and dfcomb allows binary and time-to-event toxicity endpoints.

In practice, many of the reviewed methods remain available only through author-provided code, downloadable programs, or code on request, which represents an important barrier to broader adoption. For context, adjacent software ecosystems are more mature for toxicity-only combination trials (e.g., BOIN and pocrm) and for single-agent Phase I/II utility-based designs (e.g., EffTox or BOIN12). However, a comparable user-friendly platform for seamless Phase I/II drug-combination designs that jointly handle toxicity, efficacy, and delayed outcomes is still lacking (39–42). Existing software also differs in scope: some packages focus primarily on dose escalation, whereas others support simulation, operating-characteristic evaluation, or protocol calibration. At present, no single platform provides a comprehensive framework that jointly accommodates toxicity, efficacy, continuous adaptation, and delayed-outcome functionality for adaptive Phase I/II combination trials.

We developed crm12Comb, an R package available from the Comprehensive R Archive Network, for Phase I/II drug-combination trials that simultaneously estimate toxicity and efficacy probabilities using a CRM-based partial-order design (37). Relative to existing packages such as pocrm and dfcomb, crm12Comb provides an accessible, self-contained workflow for combination CRM designs. However, it does not currently accommodate delayed or late-onset outcomes. Therefore, crm12Comb should be viewed as one specialized implementation rather than a comprehensive solution for all adaptive Phase I/II combination designs.

## 6 Discussion

This review shows that seamless Phase I/II combination designs have evolved along several distinct methodological pathways: model-based approaches that rely on monotonicity or partial ordering, utility-based or trade-off-based designs that explicitly optimize benefit-risk, and continuous-dose methods that search along an estimated maximum tolerated dose curve. No single design is uniformly preferable across all settings. Instead, design choice should reflect the clinical setting, the plausibility of monotonicity assumptions, whether the target is the MTD or an optimal biological dose, and the degree to which investigators need transparent pre-tabulated rules versus flexible model updating.

For discrete combination grids with a manageable number of dose pairs and credible monotonicity or partial-order assumptions, CRM- or copula-type designs remain attractive because they use accumulating data efficiently. However, dose-grid size directly affects feasibility since larger grids may require restricting admissible combinations, using prior ordering information, or pruning unpromising regions. Utility-based designs are especially appealing when the optimal regimen may not coincide with the MTD, as may occur with targeted therapy or immunotherapy. These approaches require investigators to quantify trade-offs between toxicity and efficacy before trial launch. This is both a strength and a barrier: it makes benefit-risk preferences explicit, but selected regimens may be sensitive to the utility matrix. Sensitivity analyses over clinically plausible utility values should therefore be part of protocol development. Continuous-dose designs are most relevant when the combination can be titrated along an estimated MTD curve, but their greater modeling and implementation burden may limit their practicality without substantial statistical support. When one agent has a well-characterized safety profile and the second is novel, prior information on the known agent may be informative, but the combination still requires careful evaluation because interaction-driven toxicity may not follow single-agent patterns.

The fact that our targeted search identified only a few clearly documented examples, all from the same research group, suggests that the major bottlenecks are not only methodological but also translational. These barriers include reliance on bespoke code, limited off-the-shelf software, clinician unfamiliarity with benefit-risk utilities, and the additional protocol work required to pre-specify allocation, stopping, and decision rules. The scarcity of real-trial implementations should therefore be interpreted as a gap between methodological development and practical implementation rather than an indication that the methods lack clinical value.

One of the most important unresolved practical challenges is the mismatch between toxicity and efficacy observation timelines. Many adaptive Phase I/II drug combination designs assume that both toxicity and efficacy outcomes are fully observed before updating randomization probabilities or selecting subsequent dose combinations. In practice, efficacy outcomes often mature later than DLT outcomes, particularly in immunotherapy settings where delayed responses and atypical toxicity profiles are common. Therefore, investigators may need to temporarily suspend adaptation, make decisions only on patients with complete outcome data, or use simplified interim rules until pending outcomes become available, all of which may reduce trial efficiency. Adjacent single-agent Phase I/II methods such as BOIN12 and TITE-BOIN12 illustrate how utility-based or time-to-event adaptations can address late-onset efficacy and toxicity, but analogous user-friendly combination-trial extensions remain limited in both methodology and publicly available software (40, 41).

Simultaneously identifying an optimal dose combination based on both toxicity and efficacy remains a central methodological challenge in adaptive Phase I/II drug-combination trials. Seamless designs can partially alleviate the information constraints of conventional early-phase development because patients enrolled after the initial dose-finding stage may continue to contribute toxicity information, and some designs allow toxicity and/or efficacy information to be shared across stages. Consequently, the effective sample size available for toxicity estimation may be larger than that of a stand-alone Phase I cohort. However, this advantage should not be overstated. The overall number of patients remains small relative to the multidimensional dose-combination space, and later-stage patients are typically concentrated among combinations already considered sufficiently safe or promising, providing limited information about other regions of the dose surface. In addition, investigators must estimate efficacy and characterize the joint benefit-risk profile rather than toxicity alone. The extent to which later-stage toxicity observations are incorporated into dose estimation also varies across designs. To achieve adequate statistical efficiency under these constraints, many methods rely on assumptions such as monotonicity, partial ordering, utility functions, parametric dose-response models, or information borrowing across dose combinations and trial stages. Although these assumptions can improve efficiency, they may reduce robustness when the assumed clinical relationships are incorrect. Future methodological research should therefore focus on designs that make efficient use of accumulating toxicity and efficacy information across stages while remaining robust to model misspecification and realistic small-sample limitations.

Regulatory guidance has helped establish adaptive designs as an acceptable component of modern drug development, but seamless combination trials require especially clear pre-specification of adaptation rules, endpoint timing, simulation operating characteristics, and stopping criteria. This is particularly important when combining agents with different toxicity mechanisms, such as cytotoxic drugs and immunotherapies, where the clinical meaning and timing of safety events may differ substantially across agents. More practical software, template protocols, and well-documented case studies may be as important for increasing adoption as additional methodological refinement. Trial protocols should report operating characteristics across clinically plausible scenarios, including the probability of selecting the true optimal regimen, probability of overdosing, the average number of patients treated at unsafe or ineffective combinations, early stopping probabilities, expected sample size, and performance under delayed-outcome scenarios. Therefore, extensive simulation studies should be conducted during trial planning to evaluate operating characteristics under plausible toxicity, efficacy, interaction, accrual, and delayed-outcome scenarios.

This review has several limitations. First, it was conducted as a structured narrative methodological review rather than a formal systematic review; accordingly, we did not perform PRISMA-style study flow reporting, risk-of-bias assessment, or quantitative synthesis. Second, by focusing on designs that simultaneously estimate toxicity and efficacy for drug combinations, we excluded adjacent areas, including Phase I combination-toxicity designs, single-agent Phase I/II methods, and sequential phase-I-then-Phase II combination programs, except where needed for context. Third, real-world implementation may be underestimated because adaptive allocation algorithms are often described incompletely in trial publications or remain embedded in unpublished protocols or industry submissions. Fourth, software availability also changes over time, and we did not independently validate each package through simulation. Finally, the reviewed designs were compared qualitatively rather than through an independent common simulation framework. Direct head-to-head comparison is challenging because these methods were developed for substantially different settings, including discrete versus continuous dose spaces, binary versus ordinal or continuous endpoints, monotonic versus non-monotonic dose-response relationships, and different optimization objectives such as utility maximization versus toxicity-constrained efficacy optimization. Consequently, numerical operating characteristics reported in the original publications are not directly comparable across methods and should be interpreted cautiously. A unified simulation framework that fairly accommodates these heterogeneous design structures would constitute a substantial methodological undertaking beyond the scope of this narrative review. Nevertheless, comparative evaluation under harmonized scenarios, potentially within subsets of designs sharing similar dose structures, endpoints, and objectives, represents an important direction for future research. More broadly, future work should prioritize robust methods for delayed outcomes, broader and more accessible software implementation, and clearer practical guidance to support investigators in selecting and implementing adaptive Phase I/II combination designs.
