# Supplementary material for: A narrative review of adaptive Phase I/II clinical trial designs for drug combinations in oncology
Source: Front Oncol. 2026 Sep 21;16:1916456. doi: 10.3389/fonc.2026.1916456 (PMC13635823; doi:10.3389/fonc.2026.1916456)
Supplement: Supplementary file 1 [file SupplementaryFile1.docx]

Supplementary Table 1: Specific adaptive methods used in different Phase I/II adaptive designs for drug combinations

| **Paper (Year)** | **Adaptive method** |
| --- | --- |
| Yin, Li, and Ji [10] (2006) | Bayesian adaptive method |
| Huang et al. [11] (2007) | Modified 3+3 for dose combinations; Response-adaptive randomization from Bayesian posterior efficacy probabilities |
| Yuan and Yin [12] (2011) | Bayesian copula-type model for Phase I dose-finding design; Response-adaptive randomization |
| Wages and Conaway [13] (2014) | CRM Phase I dose-finding design; Response-adaptive randomization |
| Thall et al. [14] (2017) | Bayesian adaptive design; Response-adaptive randomization |
| Mozgunov and Jaki [15] (2019) | Novel efficacy-toxicity trade-off function; Response-adaptive randomization |
| Mozgunov and Jaki [16] (2019) | Novel efficacy-toxicity trade-off function; Response-adaptive randomization |
| Tighiouart [17] (2019) | EWOC dose-finding design; Bayesian adaptive design; Response-adaptive randomization |
| Jiménez and Tighiouart [18] (2022) | EWOC dose-finding design; Response-adaptive randomization |
| Jiménez and Zheng [19] (2023) | EWOC dose-finding design; Bayesian adaptive method |
| Wang et al. [20] (2023) | Bayesian adaptive model selection |

Supplementary file: Technical details for two designs using continuous dose combinations

This part provides the complete statistical formulation for the Jiménez and Tighiouart [18] and Jiménez and Zheng [19] methods summarized in 3.2 Method Overview - Continuous Dose Combination Methods.docx. It retains the toxicity and efficacy models, the MTD-curve expression, prior and posterior specifications, hypothesis testing and dose-selection rules, and early-stopping criteria.

Jiménez and Tighiouart [18] refined the two-stage Phase I/II design for combinations of two cytotoxic agents with continuous dose levels. They incorporated the assumption that a single patient population could span both phases. In Phase I, they implement the EWOC method to obtain the estimated MTD curve and use response-adaptive randomization to assign patients according to the MTD curve. The doses $x, y,$were standardized to the [0, 1] interval using the dose ranges of each drug first. Let $Z$ and $E$ denote the binary outcomes of DLT and treatment response, with 1 indicating the presence of DLT or a positive response. Similar to the settings used in Tighiouart [17], the target probability of DLT,$\theta_{Z}$ and efficacy of the standard treatment, $\theta_{E}$ need to be prespecified (e.g. $\theta_{Z}=0.33$ and$\theta_{E}=0.15$ used in the simulation studies [18]). The goal is to find a drug combination $\left( x,y \right)$ such that its DLT probability $\pi_{Z}\left( x,y \right)\leq\theta_{Z}$ and its treatment efficacy $\pi_{E}\left( x,y \right)\geq\theta_{E}$. Under the assumption of a monotonically increasing relationship for both toxicity and efficacy, the optimal dose belongs to the MTD curve $\mathcal{M=}\left( x, y \right):\pi_{Z}\left( x, y \right)=\theta_{Z}$. The marginal probabilities of DLT and efficacy are defined as

$\pi_{Z}\left( x, y \right)=P\left( Z=1 | x, y \right)=F\left( \alpha_{0}+\alpha_{1}x+\alpha_{2}y+\alpha_{3}xy \right)$,

and

$\pi_{E}\left( x, y \right)=P\left( E=1 | x, y \right)=F\left( \beta_{0}+\beta_{1}x+\beta_{2}y+\beta_{3}xy+\beta_{4}x^{2}+\beta_{5}y^{2} \right)$,

where $\alpha_{1}, \alpha_{2}, \alpha_{3}, \beta_{1}, \beta_{2}, \beta_{3}, \beta_{4}, \beta_{5}>0$ and $F\left( \cdot\right)$ denotes the CDF of the standard normal distribution. The quadratic terms are included in the efficacy model to account for the complex MTD curve condition. Let $\rho_{uv}, u, v=0, 1$ be the DLT probability for dose levels $X = u$ and $Y = v$, then $\alpha_{0}=F^{-1}\left( \rho_{00} \right), \alpha_{1}=F^{-1}\left( \rho_{10} \right)-F^{-1}\left( \rho_{00} \right),$and $\alpha_{2}=F^{-1}\left( \rho_{01} \right)-F^{-1}\left( \rho_{00} \right)$, so the MTD curve can be denoted as

$\mathcal{M=}\left\{ \left( x, y \right): y=\frac{\left( F^{-1}\left( \theta\right)-F^{-1}\left( \rho_{00} \right) \right)-\left( F^{-1}\left( \rho_{10} \right)-F^{-1}\left( \rho_{00} \right) \right)x}{\left( F^{-1}\left( \rho_{01} \right)-F^{-1}\left( \rho_{00} \right) \right)+\alpha_{3}x} \right\}$,

where $\rho_{01}, \rho_{10}, \alpha_{3}$ are independent and the prior distributions and are set as $\rho_{01}\sim beta\left( 1,1 \right)$,$\rho_{10}\sim beta\left( 1,1 \right),\alpha_{3}\sim Gamma\left( 0.1,0.1 \right)$ and $\rho_{00}/\mathrm{mi}n \left( \rho_{01},\rho_{10} \right)\sim beta\left( 1,1 \right)$, conditional on $\left( \rho_{01},\rho_{10} \right)$. Let $D_{n}=\left\{ \left( x_{i}, y_{i}, Z_{i}, E_{i} \right), i=1,\ldots,n \right\}$ be the current accumulated data, then the posterior distribution of the toxicity model $\pi\left( \rho_{00},\rho_{10},\rho_{01},\rho_{11} | D_{n} \right)$ and the posterior distribution of the efficacy model ${\pi(\beta_{0}, \beta}_{1}, \beta_{2}, \beta_{3}, \beta_{4}, \beta_{5}\left| D_{n} \right)$ can be calculated under a Bayesian inference framework. Denote the number of enrolled patients as $N_{1}=C_{1}\times m_{1}$ for Phase I and $N_{2}=C_{2}\times m_{2}$ for Phase II, where $C_{1}$ and $C_{2}$ are the numbers of cohorts and $m_{1}$ and $m_{2} are$the numbers of patients in each cohort. So, the study includes a total of ${N=N}_{1}+N_{2}$ patients, based on which the MTD curve ${\hat{\mathcal{M}}}_{N}$ can be estimated. At the end of stage 2, the following hypotheses could be tested.

$H_{0}: \pi_{E}\left( x, y \right)\leq\theta_{E}$ for all $\left( x,y \right)\in{\hat{\mathcal{M}}}_{N}$ vs. $H_{1}: \pi_{E}\left( x, y \right)> \theta_{E}$ $\exists\left( x,y \right)\in{\hat{\mathcal{M}}}_{N}$.

The null hypothesis $H_{0}$ is rejected if $\max_{\left( x,y \right)\in{\hat{\mathcal{M}}}_{N}}Pr\left( \pi_{E}\left( x, y \right)> \theta_{E} | D_{N} \right)>\delta_{u}$, where $\delta_{u}$ is prespecified. The optimal dose combination selected for the following Phase II trials is then $\left( x,y \right)_{opt}=\mathrm{argmax}_{\left( x,y \right)\in{\hat{\mathcal{M}}}_{N}}Pr\left( \pi_{E}\left( x, y \right)> \theta_{E} | D_{N} \right)$. Early stopping rules for both futility and safety have also been considered. The trial can be stopped early for futility when

$\max_{\left( x,y \right)\in\hat{M}} [Pr(F\left( \beta_{0}+\beta_{1}x+\beta_{2}y+\beta_{3}xy+\beta_{4}x^{2}+\beta_{5}y^{2} \right)>\theta_{E}|D_{N})]< \delta_{0}$,

where $\delta_{0}$ is a prespecified threshold. For safety concerns, the trial can be stopped early in Phase I if $\Pr\left( \pi_{Z}\left( x=0, y=0 \right)>\theta_{Z}+0.1 | D_{n} \right)>\delta_{\theta_{1}}$ with prespecified $\delta_{\theta_{1}}=0.5$, or in Phase II if $\Pr\left( \Theta>\theta_{Z}+0.1 | D_{n} \right)>\delta_{\theta_{2}}$ with prespecified $\delta_{\theta_{2}}=0.7$. A non-informative Jeffreys prior $beta\left( 0.5,0.5 \right)$ can be applied to the DLT rate $\Theta$ in both phases regardless of dose combinations.

Jiménez and Zheng [19] modified the efficacy probability of Jiménez and Tighiouart [18] to be $P\left( E=1 | x,y \right)=F\left( \beta_{0S}+exp\left( \beta_{1S} \right)x+exp\left( \beta_{2S} \right)y+exp\left( \beta_{3S} \right)xy \right)$, where $S = 1, 2$ denotes the two phases, $E=1$ indicates the occurrence of the efficacy event, $\beta_{0}$ is the efficacy probability at the lowest dose combination, $\beta_{1}$ and $\beta_{2}$ determine the contributions of two individual drugs to the overall efficacy probability, and $\beta_{3}$ captures any additional increase in efficacy probability due to the drug-drug interaction. A robust Bayesian hierarchical model has been implemented to combine efficacy data across two phases, which improves the precision of parameter estimation and reduces the number of patients treated at subtherapeutic dose combinations.
